# A systematic approach to patient safety and surgical education during a surgical mission in a resource-limited setting: A case report of double free flap reconstruction for a lower facial defect^✰^

**DOI:** 10.1016/j.jpra.2026.04.003

**Published:** 2026-04-15

**Authors:** Daniel Önefäldt, Enku Shiferaw Belayneh, Abeje Brhnu Menjeta, Andres Rodriguez Lorenzo

**Affiliations:** a Department of Plastic and Maxillofacial Surgery, Uppsala University Hospital, Uppsala, Sweden; b Department of Surgical Sciences, Uppsala University, Uppsala, Sweden; c Department of Plastic surgery, St Paul Hospital, Addis Ababa, Ethiopia

**Keywords:** Free flap, Microsurgery, Teaching, Resource-limited setting, Case report

## Abstract

Reconstruction of complex mandibular and lower facial defects poses significant challenges, especially in resource-limited settings. Double free flap reconstruction offers tailored tissue replacement when single-flap options are inadequate. We report the case of a 25-year-old male with a large composite mandibular and oral floor defect following a failed pedicled flap reconstruction after gunshot trauma. A structured checklist was used for planning and executing a safe and educational microsurgical procedure in a resource-limited setting. The patient underwent successful reconstruction using a chimeric osteocutaneous free fibula flap for mandibular framework and oral lining, combined with a free anterolateral thigh flap for external soft tissue and lower lip reconstruction, including a tensor fascia lata sling for lip suspension. Complex double free flap reconstruction can be safely achieved in low-resource environments through meticulous planning, multidisciplinary teamwork, and adaptation to available resources, providing functional and aesthetic outcomes. The presented checklist provides a practical framework for planning similar cases that balances patient safety with surgical education.

## Introduction

Reconstructive microsurgery has advanced considerably, but specialized training in advanced head and neck reconstruction is scarcely available in low-income countries (LIC) compared to high-income countries (HIC), despite the significant progress in reconstructive microsurgery across sub-Saharan African countries.^1^ One of the risks associated with performing complex microsurgical procedures during short-term international missions is the potential for increased complications and compromised patient safety, given the limited duration of the visiting team’s presence.^2^ Therefore, it is essential to establish a systematic approach that prioritizes both patient safety and the structured transfer of surgical competencies to local teams.^3^

In this report, the authors describe their experience implementing a systematic approach to patient safety and surgical education during a microsurgical mission in Ethiopia. Using a double free flap reconstruction case as an example, the report highlights key considerations for ensuring patient safety and effective surgical education, and proposes a checklist to guide future missions. This case report has been reported in line with the SCARE and STROBE guidelines.^4^^,^^5^

## Case report

A 25-year-old male soldier presented 30 months after sustaining a high-velocity gunshot wound to the lower face during armed conflict. A reconstruction attempt using a pedicled pectoralis major muscle flap was carried out but ultimately failed. At the time of presentation to our unit, the patient reported significant difficulty in feeding, persistent drooling, and impaired speech articulation. Additionally, he experienced notable weight loss due to feeding difficulties and expressed psychosocial distress related to facial disfigurement, which he attempted to conceal by wearing a mask. He had no history of smoking.

**Figure 1:**
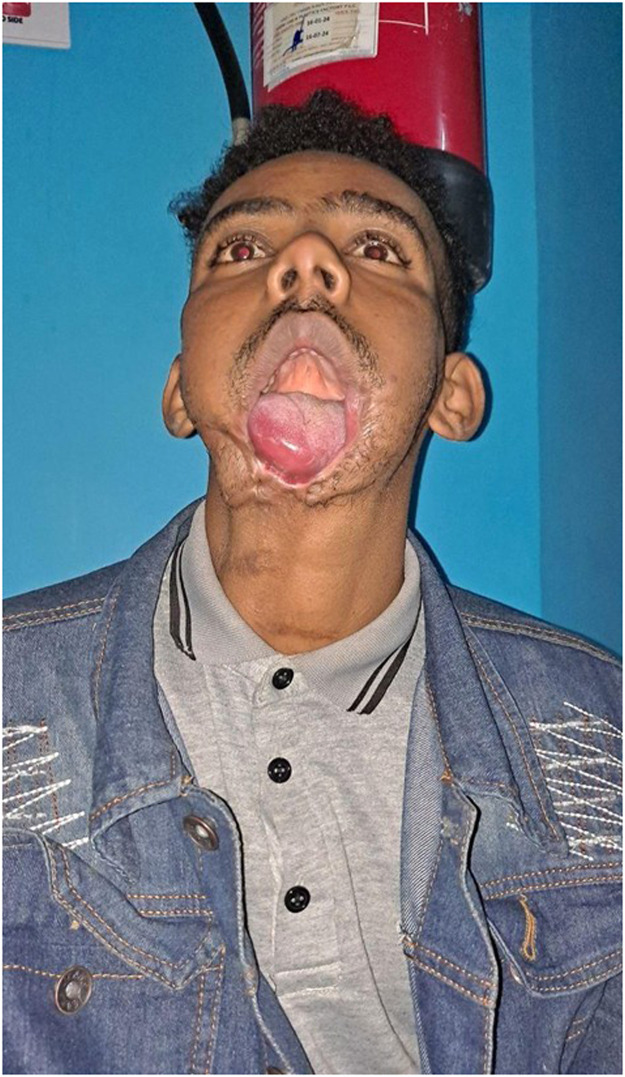
Pre-operative evaluation showed significant lower face defect with absent anterior mandible, lower lip and mentum, with significant tongue ptosis resulting in inability to close mouth, drooling and difficulty with oral nutrition.

The patient exhibited a composite lower facial defect, including total loss of the lower lip, chin, anterior floor of the mouth, and mandible from angle to angle. The tongue was prolapsed and tethered to the neck by dense scar tissue, contributing to functional impairment and continuous drooling (Figure 1, Supplemental Figures S1–2). Computed tomography imaging of the face and neck demonstrated a mandibular defect extending from angle to angle, with well-opacified bilateral cervical vessels (Supplemental Figure S3). Following multidisciplinary evaluation and discussion by the plastic and maxillofacial surgery teams, a surgical plan was proposed involving reconstruction with a double free flap: a free fibula osteocutaneous flap (FFF) for mandibular reconstruction and oral lining and a free anterolateral thigh (ALT) flap for external skin coverage, incorporating a tensor fascia lata sling for lower lip suspension to restore oral competence. The procedure was performed at Saint Paul’s Hospital Millennium Medical College in Addis Ababa, which has operating theater capacity and intensive care facilities. For instrumentation, an ophthalmological microscope was used. Microsutures and micro-instruments were donated by Operation Smile, and the osteotomies were performed using the locally available equipment, including a Gigli saw, cerclage wires, and miniplates with screws.

A structured framework was applied throughout the mission to ensure patient safety and facilitate skill transfer. The detailed steps of this approach are summarized in Table 1.

**Table 1:** Checklist for patient safety in a microsurgical mission in a low-resource setting.

Checklist	Details
**Online screening** **(Weeks before mission):**	Risk/benefit/resource assessment by local and international teams. Conducted jointly by the local and international teams to identify potential risks, assess benefits, and evaluate available resources.
**Onsite patient evaluation**	Performed by the surgical team to provide final information to the patient, assess clinical status, and evaluate vascular supply of the flaps using manual Doppler. Final patient briefing, clinical exam, Doppler assessment of perforators and recipient vessels.
**Microsurgical equipment check**	Online and onsite confirmation of the availability and functionality of the operating microscope, microsurgical instruments, and adjuncts such as heparin and lidocaine.
**Postoperative care check**	**Tracheostomy management:** Ensure 24/7 nursing staff competence in tracheostomy care. **Flap monitoring:** Regular assessment of flap viability. **Intensive care availability:** Confirm intensive care access for the first 72 h postoperatively. **Reoperation plan:** Prepare for backup surgery if flap viability is compromised within the first 72 h. **Nursing education:** International nursing team provides preoperative training (lecture and demonstration) and postoperative support for flap monitoring. **Medication:** Ensure availability of antibiotics, thrombosis prophylaxis, adequate analgesic agents and means of parenteral/enteral nutrition.
**Intraoperative planning check**	Develop a detailed surgical scheme outlining procedural steps and team division. Define the lead surgeon and organize two teams based on experience, combining international and local surgeons for flap harvesting, anastomosis, donor site closure, and flap inset. Clearly assign roles to each team member preoperatively. Scrub nurse supervision by an international scrub nurse ensures optimal workflow and competence.
**Post-mission continuity**	Backup plan for care after international team departure.

**Figure 2:**
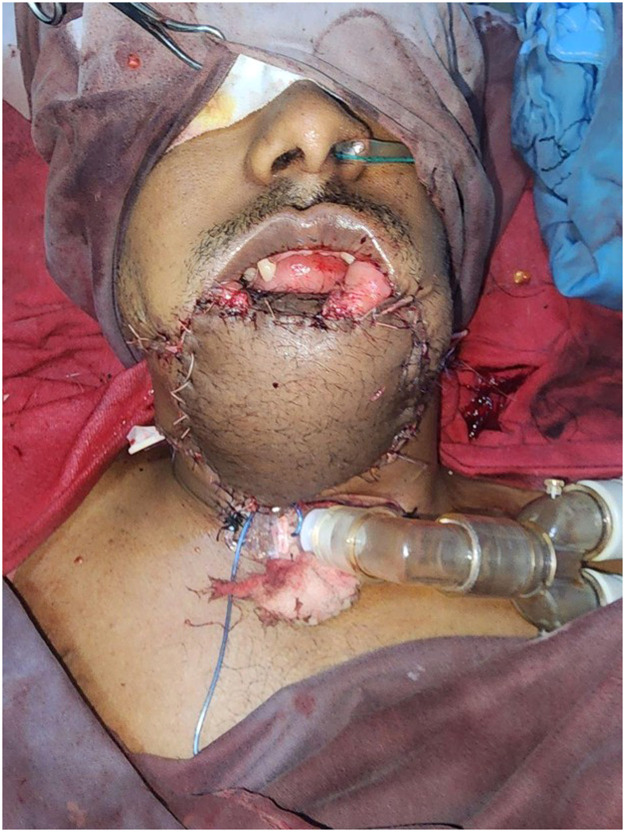
Three months post-op the patient had good flap healing with adequate mouth opening, oral seal and aesthetically acceptable lower face.

Using a two-team approach, a right osteocutaneous FFF with chimeric skin paddle was harvested and contoured for mandibular reconstruction and anastomosed to recipient right superior thyroid artery and facial vein (Figure 2, Supplemental Figures S4–7), while an ALT flap was harvested for external coverage and lower-lip reconstruction supported by a tensor fascia lata sling and anastomosed to recipient left lingual artery and facial vein (Supplemental Figure S8).

**Figure 3:**
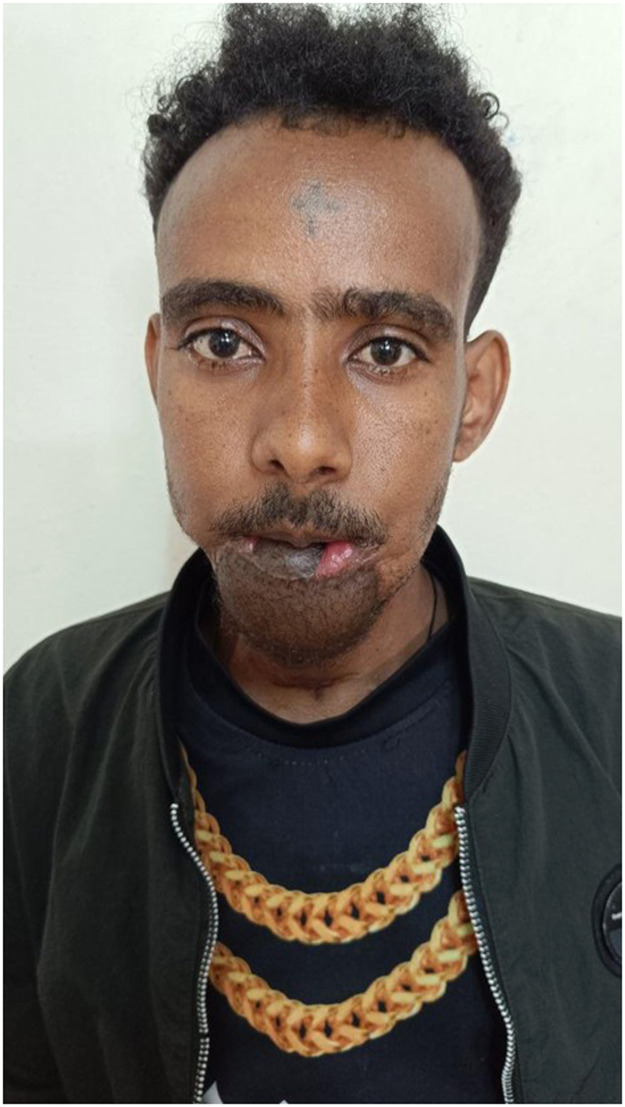
Intraoperative photo at the end of the surgery after insetting of Fibula and Anterolateral thigh flaps.

The procedure lasted 14 h without any need for transfusion. The patient was monitored in the ICU for 72 h with standardized flap checks and tracheostomy care, then discharged on postoperative day 27. Oral rehabilitation was initiated on postoperative day 10 with gradual mobilization of the mandible. Recovery was uneventful, with good oral competence and satisfactory aesthetics at 3-month follow-up (Figure 3). Following the post-mission continuity plan, the patient underwent secondary surgery performed independently by the local team after online consultation with the international team 4 months after the double free flap surgery. The procedure consisted of debulking of the ALT flap to improve the aesthetic outcome as well as speech and food intake (Supplemental Figure 9).

## Discussion

This case demonstrates that complex double free flap reconstruction can be safely performed in a LIC when supported by structured planning. The checklist (Table 1) provided a framework for case selection, intraoperative coordination, and postoperative monitoring, supporting both patient safety and local skill development. Using available resources, the team achieved functional and aesthetic reconstruction without complications.

The FFF remains the gold standard for long mandibular reconstruction, and extensive composite defects may require double free flaps for adequate tissue replacement.^6^ While the technical aspects of such procedures are well established in HIC, their implementation in low-resource settings remains limited by infrastructure, training, and postoperative care capacity.^7^ This case supports existing evidence that complex microsurgical reconstruction can be successfully introduced in LIC when patient selection is appropriate and structured perioperative systems are in place.^8^

### Patient safety and educational framework

The checklist presented in Table 1 was central to minimizing risk and structuring the educational component of the mission. It addressed preoperative, intraoperative, and postoperative phases with defined responsibilities and redundancy planning. The framework supported both safe execution and real-time skill transfer. Patient safety was further reinforced through standardized ICU monitoring, tracheostomy care, and the establishment of a reoperation plan. The inclusion of postoperative training for nursing staff is essential for reliable flap monitoring and early complication recognition.^9^ These measures demonstrate that adherence to structured safety checklists can offset some of the inherent risks of complex microsurgery in LIC.^10^

### Future implications and take-away lessons

This case suggests that advanced reconstruction in LIC depends less on technology than on preparation, structured teamwork, and shared responsibility between visiting and local teams. The checklist presented here offers a simple and reproducible framework to guide each phase of care, helping teams anticipate risks while creating structured teaching opportunities. One of the important aspects of patient safety is the proper selection of indications for complex surgery in a low-resource setting, where the risk–benefit balance must be clearly justified. For this patient, a thorough discussion was conducted before the surgical mission, concluding that the expected benefits outweighed the risks. The patient was 24 years old, and no locoregional options were available that could provide a satisfactory functional or aesthetic reconstruction for such a large and complex defect. Attempting an additional local option would have only increased morbidity without offering meaningful improvement in oral function or appearance.

By combining patient safety measures with progressive skill transfer, complex microsurgery can be both safe and educational. Future collaborations should emphasize continuity and mentorship, allowing local surgeons to gradually assume greater responsibility as experience and resources expand, thereby supporting sustainable capacity building in settings where the need for advanced reconstruction remains high.

## Conclusion

Complex lower face reconstruction is feasible and effective even in resource-limited settings with adequate planning. The combined use of a FFF and an ALT flap provided structural, functional, and aesthetic reconstruction. Careful preoperative planning and coordinated multidisciplinary execution using a checklist were critical to the success of the procedure. This approach can serve as a model and inspiration for implementation of advanced reconstructive care in similarly resource-constrained environments.

## Informed consent, ethical approval and compliance

This manuscript describes a single clinical case and does not involve experimental research. All procedures were carried out in accordance with relevant Ethiopian laws and institutional guidelines and in compliance with the principles of the Declaration of Helsinki. Formal ethical committee approval was not required according to institutional policy. Oral informed consent was obtained from the patient prior to surgery. Written informed consent was obtained for inclusion of clinical information in this case report and for publication of clinical photographs, including identifiable facial images, in an open-access journal. The patient was informed about the purpose and public accessibility of the publication. Patient privacy and confidentiality were otherwise respected. This case report was prepared in accordance with the SCARE guidelines, and relevant items from the STROBE statement were considered where applicable.

## Declaration of generative AI and AI-assisted technologies in the manuscript preparation process

During the preparation of this article the authors used ChatGPT 5.2 in order to improve the readability of the manuscript. After using this tool, the authors reviewed and edited the content as needed and take full responsibility for the content of the publication.

## Author contributions

All authors participated in the surgery and care described in this case report. All authors participated in writing and editing the manuscript and have approved the final version for publication.

## Declaration of competing interest

The authors declare the following conflict of interest: This case was part of a Visiting Professorship in Microsurgery to St Paul Hospital in Addis Ababa organized by Operation Smile Sweden and Operation Smile Ethiopia. Operation Smile covered expenses for the trip for authors ARL and DÖ, who received no other financial compensation.

## Appendix Supplementary materials

Supplemental Figure S1: Pre-operative evaluation showed significant lower face defect with absent anterior mandible, lower lip and mentum, with significant tongue ptosis. Image, image 1 dummy alt text

Supplemental Figure S2: Pre-operative evaluation showed significant lower face defect with absent anterior mandible, lower lip and mentum, with significant tongue ptosis. Image, image 2 dummy alt text

Supplemental Figure S3: Computer tomography 3D reconstruction showed an angle-to-angle mandibular defect. Image, image 3 dummy alt text

Supplemental Figure S4: Pre-operative marking for a chimeric free fibula with skin paddle. Image, image 4 dummy alt text

Supplemental Figure S5: A plastic ruler was used as template to section the fibula in three parts before molding to a mandibular shape. Image, image 5 dummy alt text

Supplemental Figure S6: Bone fragments were fixed with miniplates and stainless-steel wire after pedicle was divided. Image, image 6 dummy alt text

Supplemental Figure S7: The fibula was fixed to the mandibular ramus with miniplates and wires, before skin paddle was inset to reconstruct the oral floor and lower lip. Image, image 7 dummy alt text

Supplemental Figure S8: A strip of tensor fascia lata was used as a sling to suspend the lower lip. Image, image 8 dummy alt text

Image, image 9 dummy alt text
