# Supplementary figures and images for: A systematic approach to patient safety and surgical education during a surgical mission in a resource-limited setting: A case report of double free flap reconstruction for a lower facial defect
Source: JPRAS Open. 2026 Apr 15;50:95–101. doi: 10.1016/j.jpra.2026.04.003 (PMC13147769; doi:10.1016/j.jpra.2026.04.003)

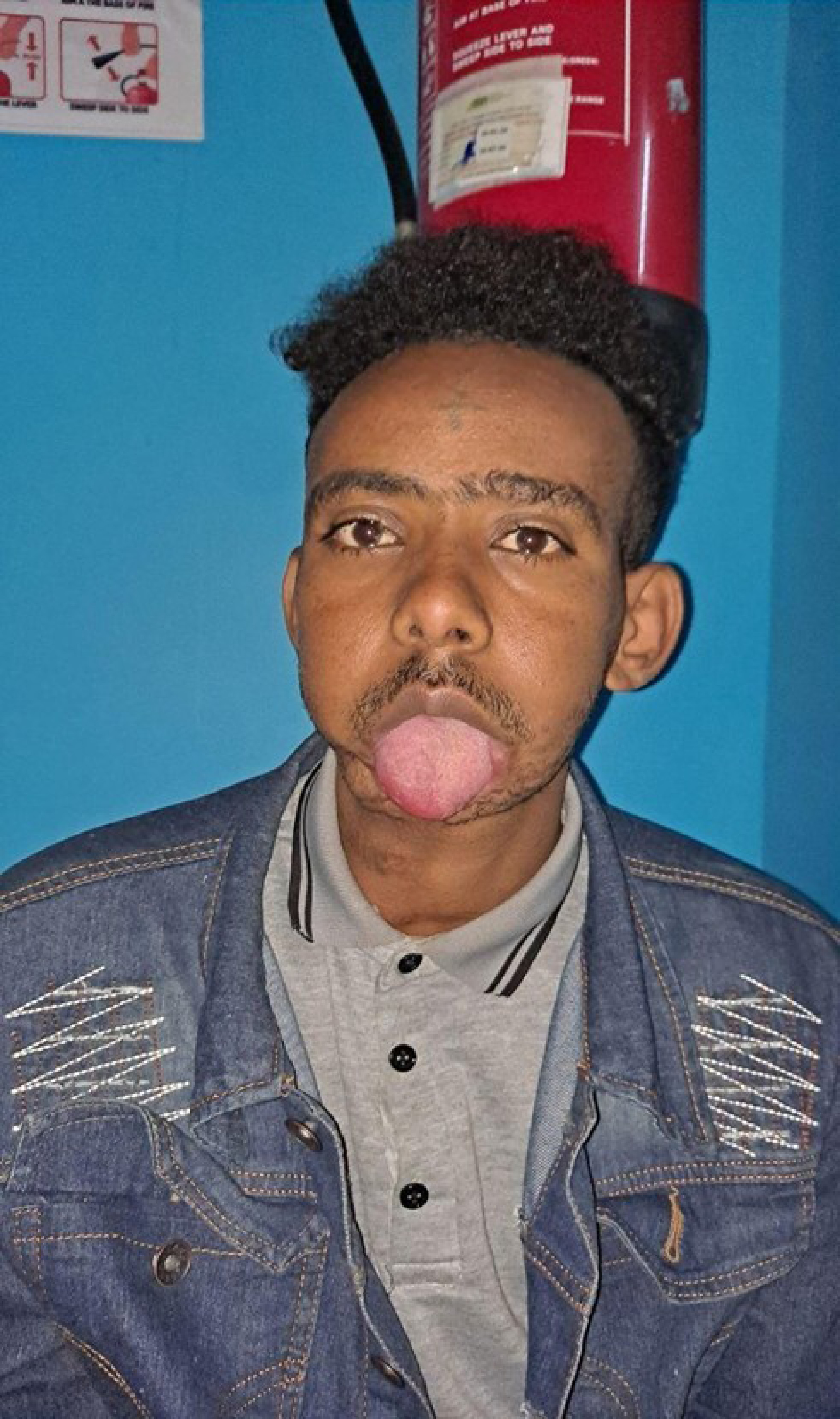

Supplement: Supplementary file 1 — Supplemental Figure S1: Pre-operative evaluation showed significant lower face defect with absent anterior mandible, lower lip and mentum, with significant tongue ptosis. [file mmc1.jpg]

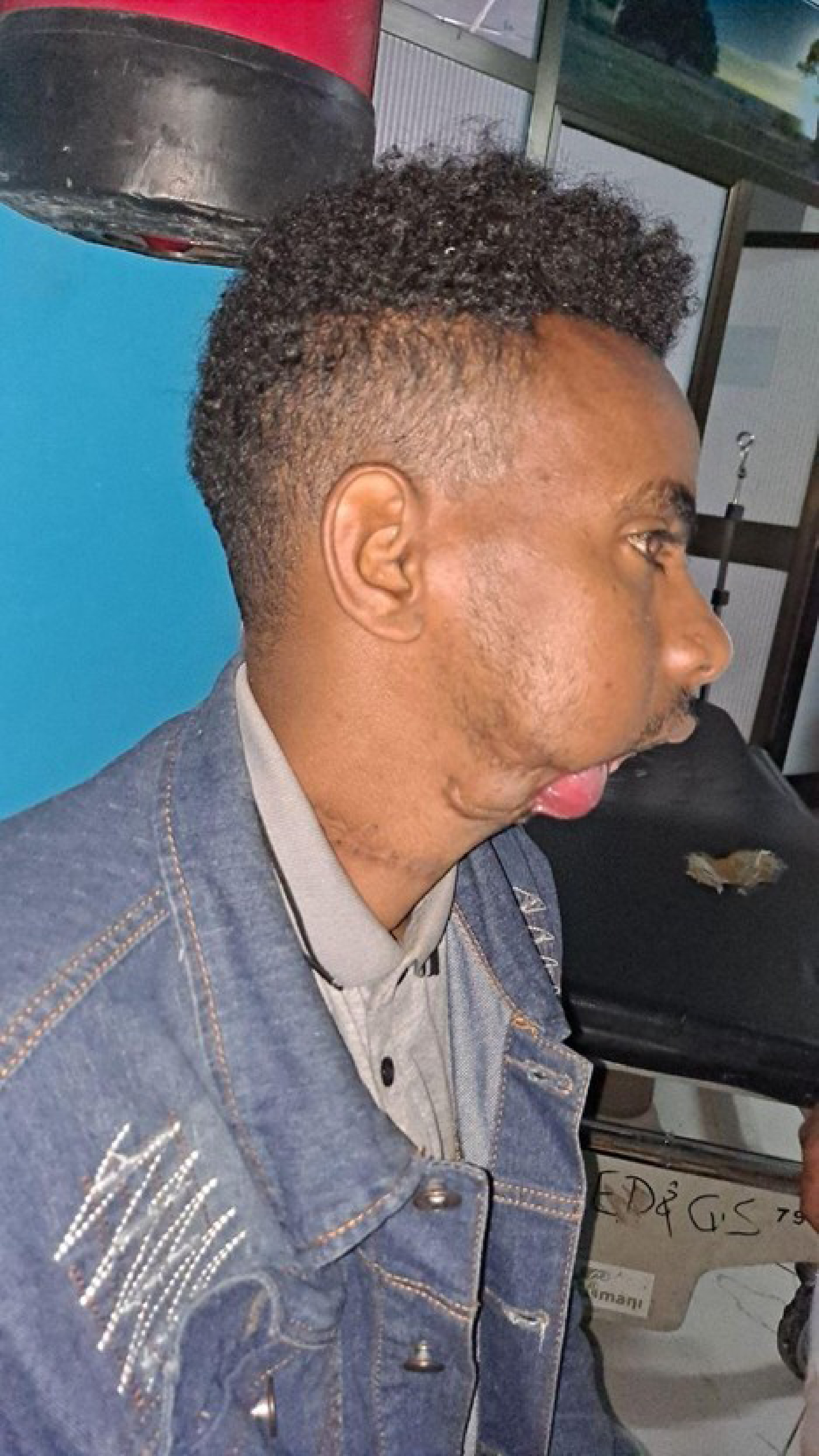

Supplement: Supplementary file 2 — Supplemental Figure S2: Pre-operative evaluation showed significant lower face defect with absent anterior mandible, lower lip and mentum, with significant tongue ptosis. [file mmc2.jpg]

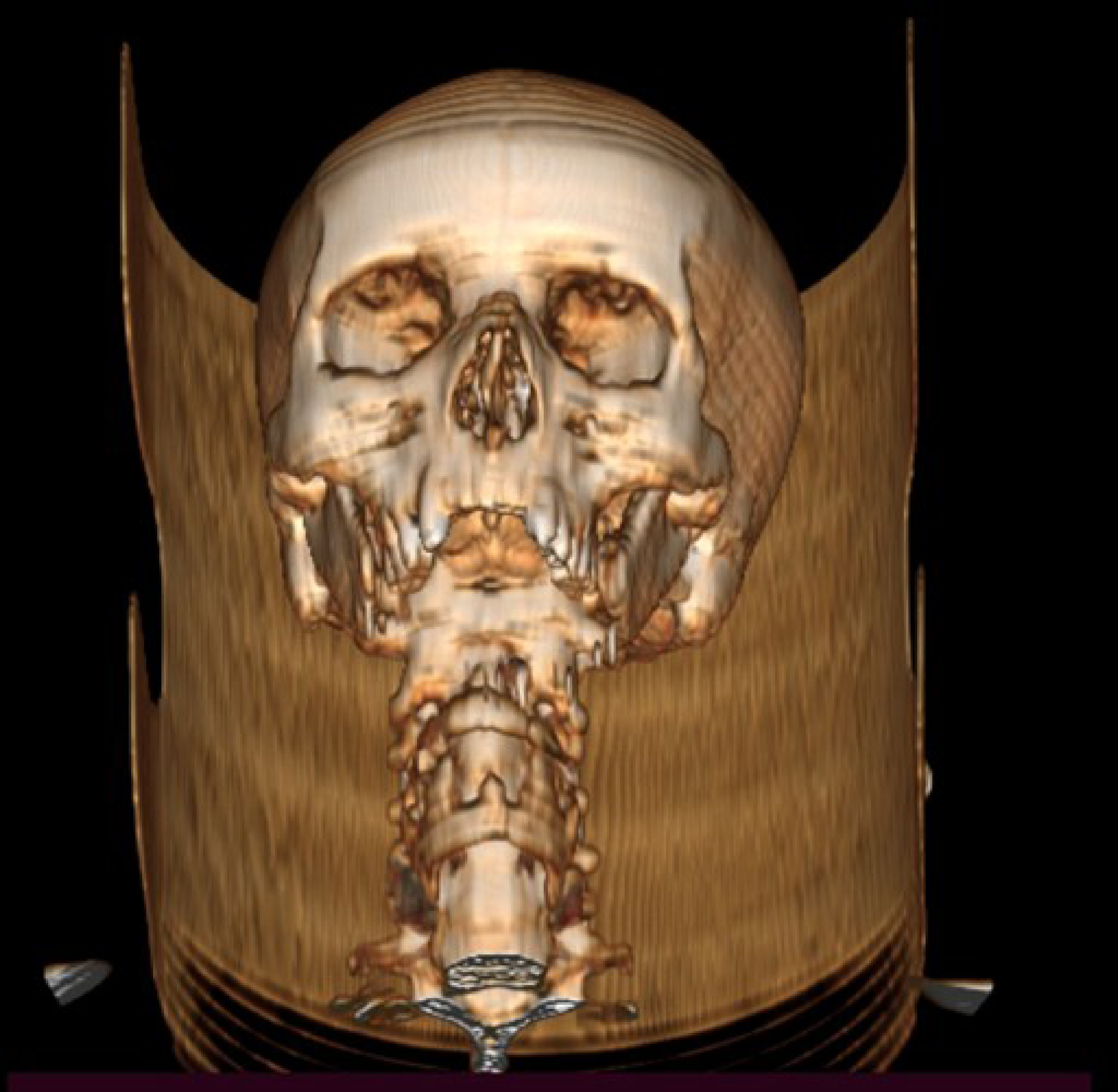

Supplement: Supplementary file 3 — Supplemental Figure S3: Computer tomography 3D reconstruction showed an angle-to-angle mandibular defect. [file mmc3.jpg]

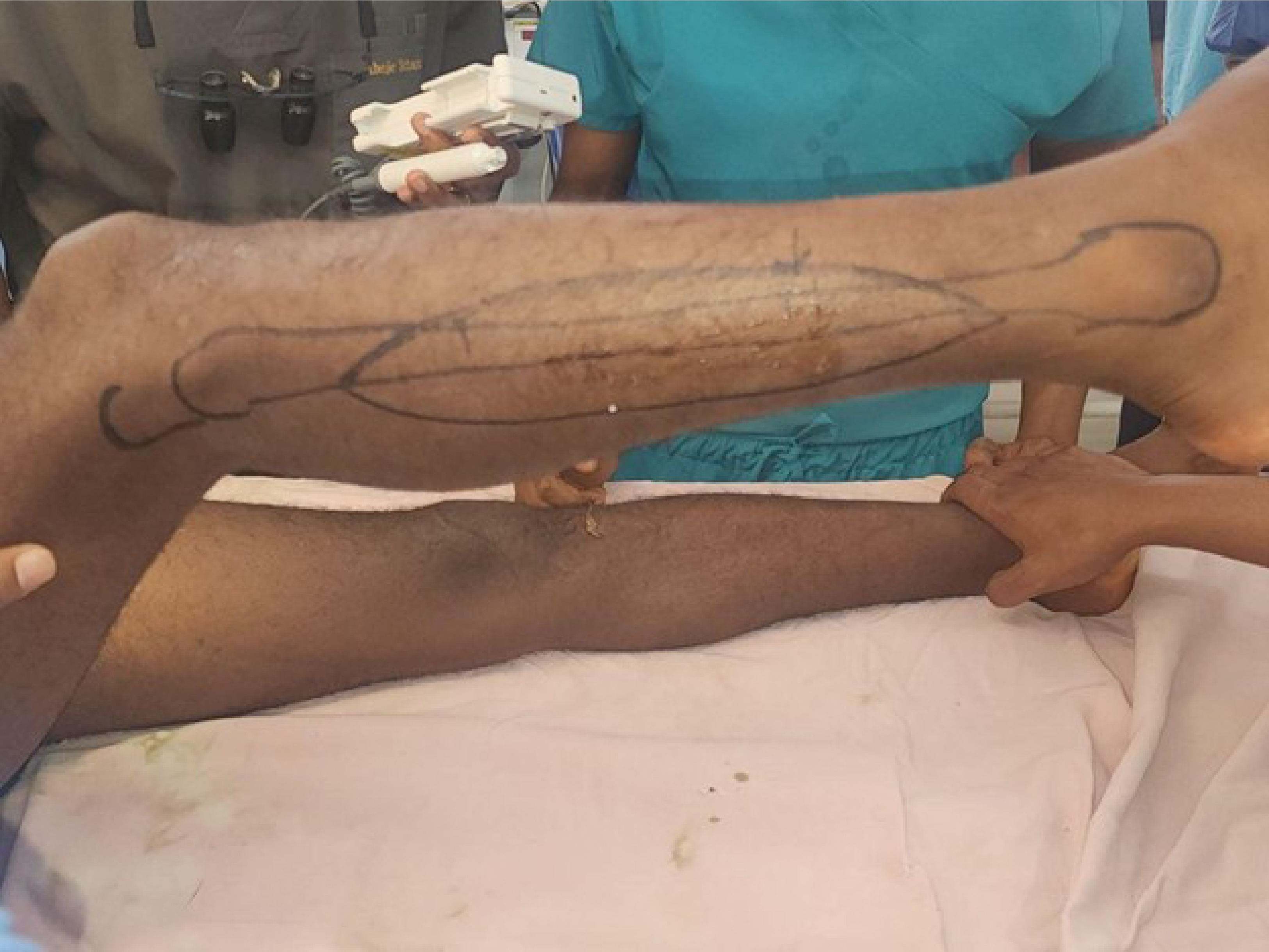

Supplement: Supplementary file 4 — Supplemental Figure S4: Pre-operative marking for a chimeric free fibula with skin paddle. [file mmc4.jpg]

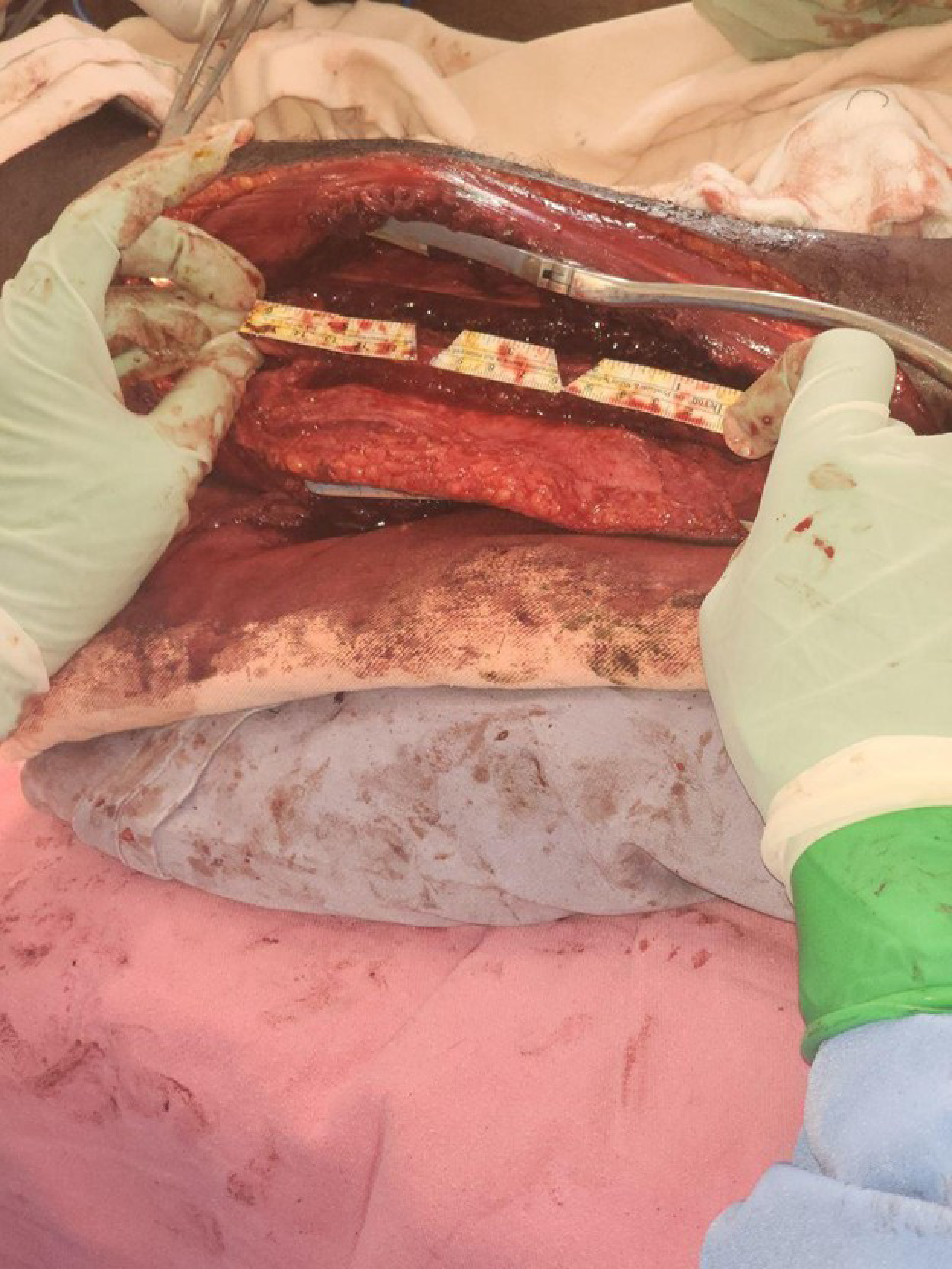

Supplement: Supplementary file 5 — Supplemental Figure S5: A plastic ruler was used as template to section the fibula in three parts before molding to a mandibular shape. [file mmc5.jpg]

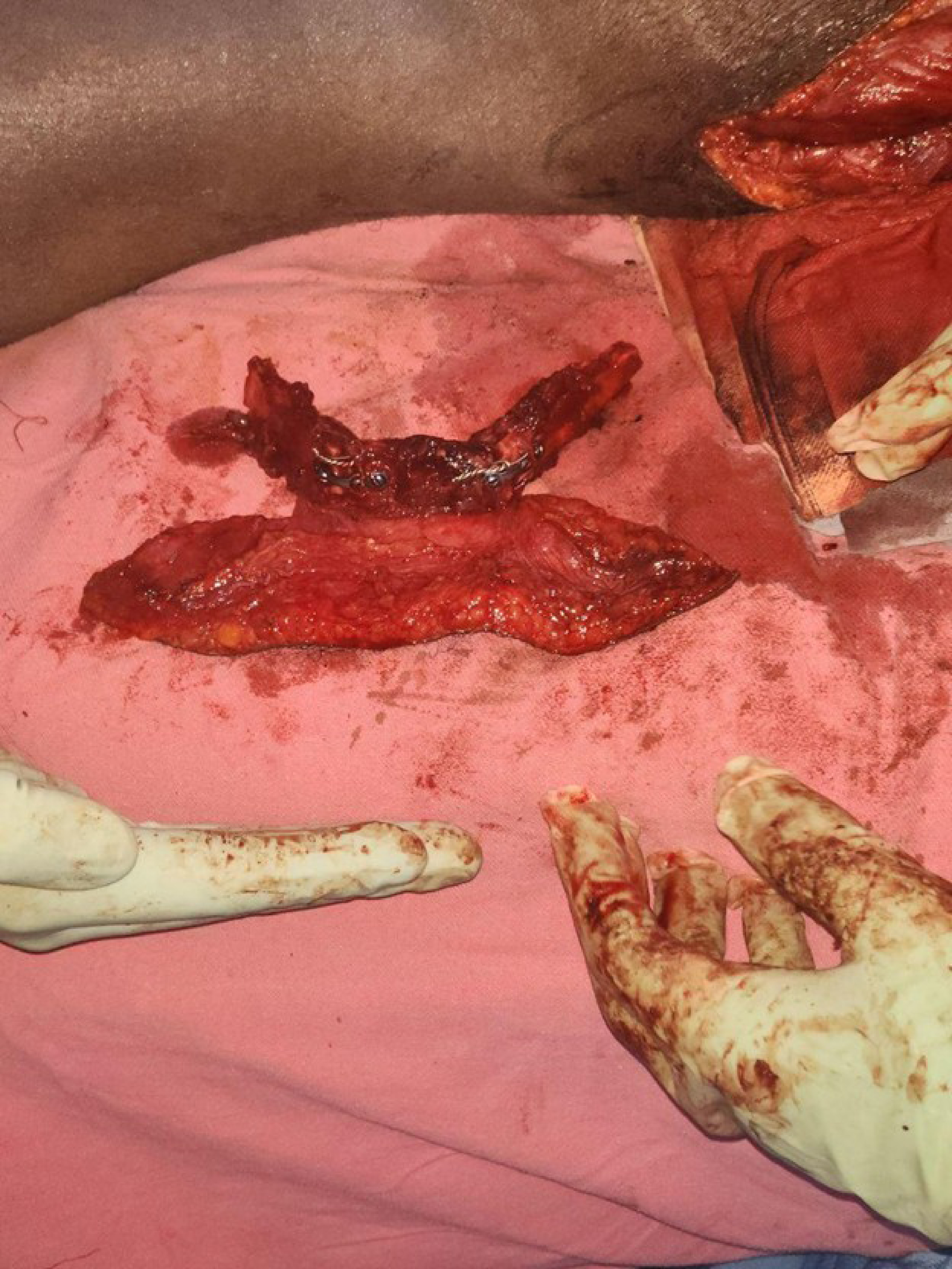

Supplement: Supplementary file 6 — Supplemental Figure S6: Bone fragments were fixed with miniplates and stainless-steel wire after pedicle was divided. [file mmc6.jpg]

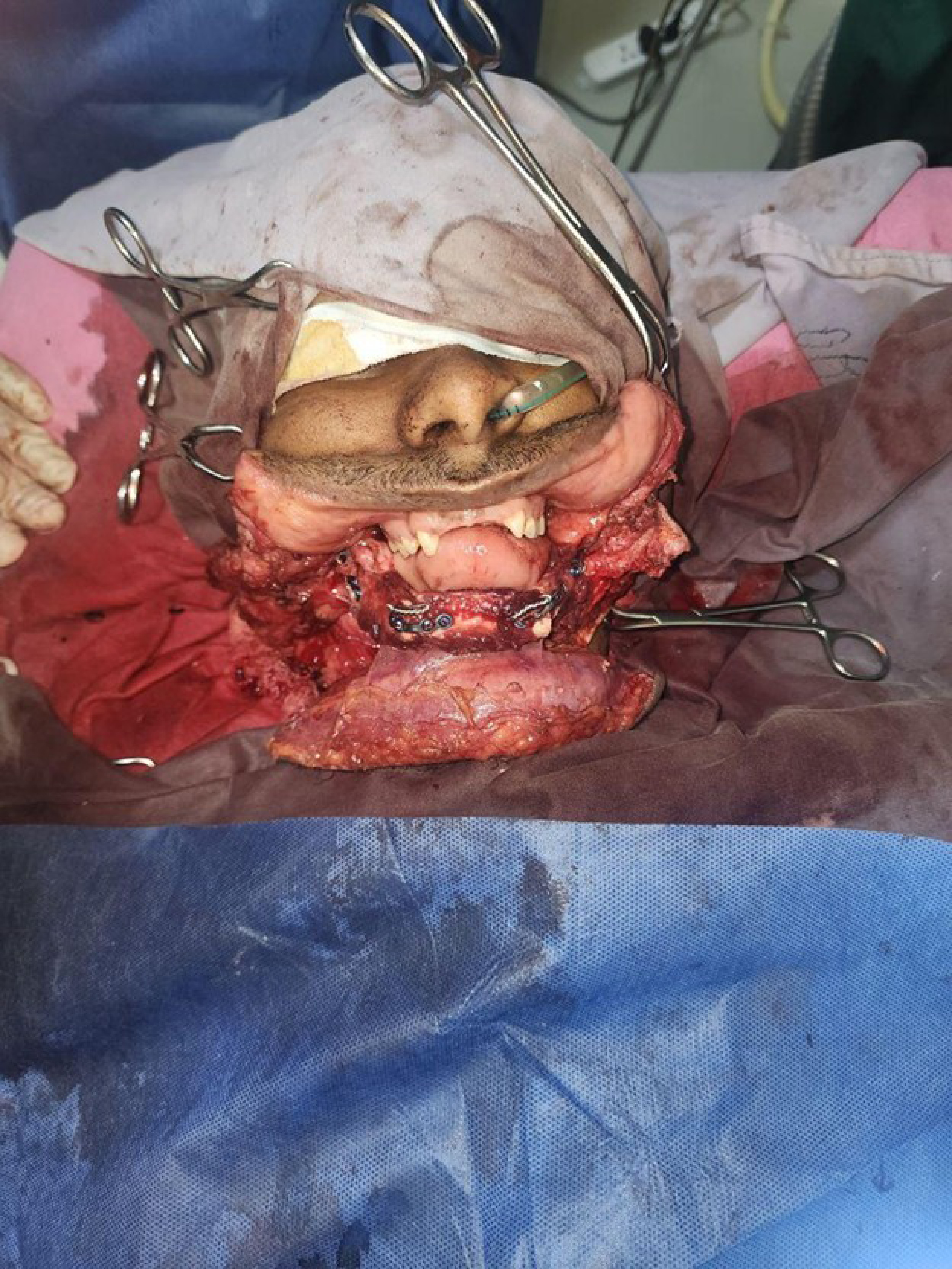

Supplement: Supplementary file 7 — Supplemental Figure S7: The fibula was fixed to the mandibular ramus with miniplates and wires, before skin paddle was inset to reconstruct the oral floor and lower lip. [file mmc7.jpg]

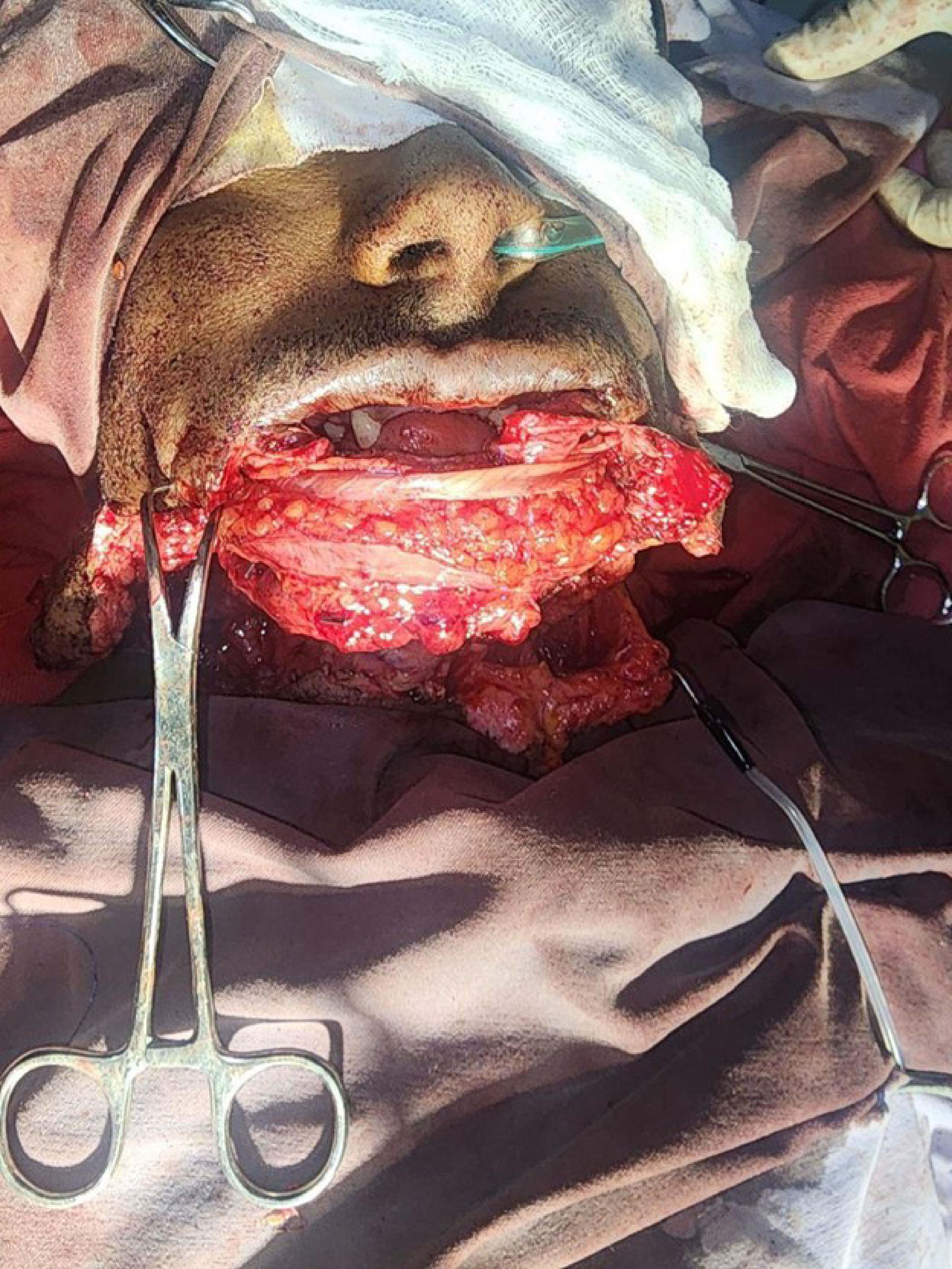

Supplement: Supplementary file 8 — Supplemental Figure S8: A strip of tensor fascia lata was used as a sling to suspend the lower lip. [file mmc8.jpg]

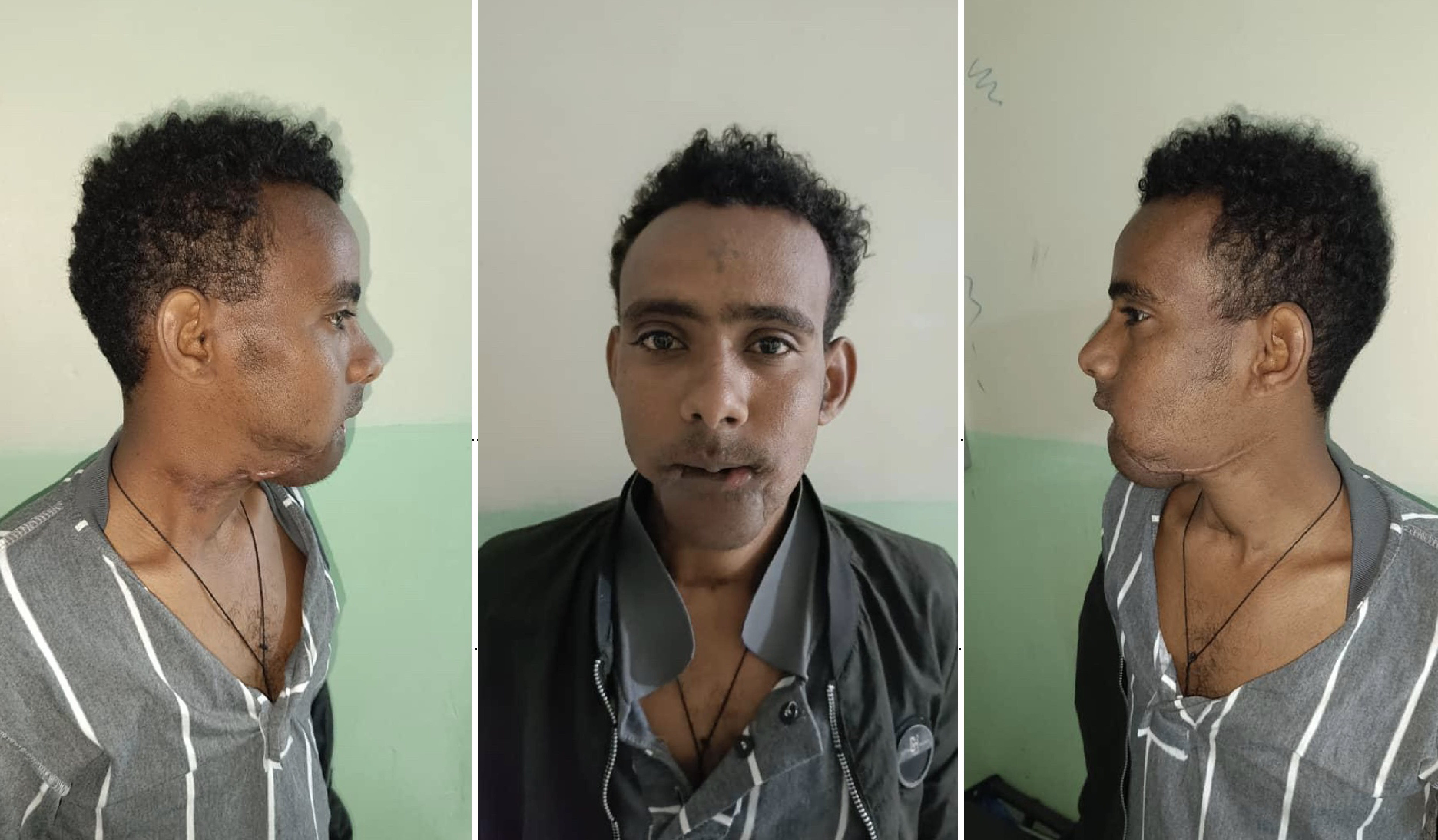

Supplement: Supplementary file 9 [file mmc9.jpg]
